# Procalcitonin as an Endogenous Biomarker for Mastitis in Cows

**DOI:** 10.3390/ani13132204

**Published:** 2023-07-05

**Authors:** Stephan Neumann, Stephan Siegert, Anneke Fischer

**Affiliations:** Institute of Veterinary Medicine, Georg-August University of Goettingen, D-37077 Goettingen, Germany; stephan.siegert@uni-goettingen.de (S.S.);

**Keywords:** mastitis, biomarker, procalcitonin, cows

## Abstract

**Simple Summary:**

The aim of the present study was to investigate a measurable biomarker for the detection and differentiation of mastitis in cows. We chose procalcitonin as a candidate because it is synthesized during inflammation of bacterial origin. We tested the marker in milk and serum in healthy cows and those with subclinical and clinical mastitis. The results show that procalcitonin is suitable for the detection and differentiation of mastitis in cows and thus can be a potential biomarker.

**Abstract:**

Mastitis is one of the most common diseases of dairy cows. Procalcitonin (PCT) has been described as an endogenous inflammatory biomarker for bacterial infections. The aim of this study was to find possible correlations between PCT concentrations in the serum and milk of cows with mastitis and their clinical signs and disease progression. In total, 88 dairy cows were examined, of which 30 animals were diagnosed with clinical mastitis, 30 had subclinical mastitis, and 28 were designated as a healthy control group. The diseased animals were re-examined after 12 days. All PCT levels in this study were determined by a species-specific ELISA. All three groups could be differentiated from each other based on serum and milk PCT levels. The animals with clinical mastitis showed the highest mean concentrations of PCT (serum: 2641 pg/mL; milk: 1326 pg/mL), and the lowest PCT concentrations were found in the healthy control group (serum: 1166 pg/mL; milk: 176 pg/m). Over the course of the disease, results from the kinetics study showed that PCT levels remained high for the entire observation period. The results from this study showed that the PCT concentration could be used to differentiate between clinical mastitis, subclinical mastitis, and healthy cows.

## 1. Introduction

Procalcitonin (PCT) is a prohormone of calcitonin. In healthy organisms, the production of PCT takes place in thyroid C cells. The production is conducted by calcitonin gene-related peptide I (CALC-1), which is located on chromosome 11. The mRNA product is called preprocalcitonin. Further modification transforms it to 116-amino-acid procalcitonin. Finally, all the PCT produced in the thyroid C cells gets converted to calcitonin. Thanks to these circumstances, generally no PCT gets released into the circulation of a healthy individual. Therefore, PCT levels in healthy individuals remain very low. During an infection, PCT is produced mainly by two different mechanisms: the direct pathway induced by lipopolysaccharide or other toxic metabolites from microbes and the indirect pathway where inflammatory mediators (e.g., IL-6, tumour necrosis factor-α (TNF-α)) induce its production [1]. This is why the serum concentration of PCT rises tremendously during bacterial infections and the levels appear to normalize just as fast once the infection is terminated. Therefore, PCT is considered an acute phase reactant. These abilities allow PCT to be considered as a potentially useful biomarker. PCT has already been examined as a possible marker of the systemic inflammatory response to infections. Numerous studies have proved its efficacy as a marker for critical illness and sepsis [2]. Therefore, in human medicine, PCT is known as a highly specific marker for microbial infections and sepsis. The measured level of PCT concentration in human serum is used to differ between patients with severe bacterial infections from those with severe but still non-septic infections. Here, PCT can serve as an important parameter to decrease unnecessary use of antibiotics. Moreover, in veterinary medicine, PCT is considered a biomarker that serves as a specific indicator of the severity of bacterial infections. PCT has already been investigated in horses, cattle, and dogs [3]. Regarding cattle, PCT is already being discussed as a useful biomarker, since PCT levels are increased in calves suffering from sepsis [4,5,6,7]. There are also raised PCT levels in feedlot calves suffering from bovine respiratory disease [8]. Further, it was shown that PCT levels seem to correspond with staphylococcal mastitis [9]. Another study showed PCT’s ability to diagnose and prognose inflammation due to bacterial infection [10]. However, there is still insufficient information on the application of PCT in veterinary medicine [11]. Mastitis is a disease of the mammary gland. It is caused by different pathogens such as bacteria, viruses, fungi, and algae. It is most frequently caused by bacteria and has manyfold aetiologies. An inflammatory response is provoked. Mastitis is classified as clinical or subclinical. While clinical mastitis results in abnormal milk production and sometimes udder alteration and/or clinical signs, the subclinical mastitis mobilizes inflammatory cells to the udder but causes no changes regarding milk or udder characteristics. Mastitis is globally a significant challenge regarding milk production. It causes tremendous financial losses to dairy farms. Costs related to this disease are caused by the reduction and/or discarding of milk, as well as animal replacement [12]. Therefore, the aims of this study are to investigate the PCT levels in cows with acute clinical and subclinical mastitis, to use PCT as a marker for diagnosing different forms of mastitis and to give information about the severity and the course of the disease, and finally to compare its value with other clinical and laboratory parameters.

## 2. Materials and Methods

### 2.1. Animals

In this prospective study, we examined a total of 88 female cows of the Holstein Friesian breed. They were classified into three groups.

Group 1 was the healthy controls. All of these cows were free of any clinical signs of a disease. This was checked with a clinical investigation and an observation period of two weeks. The leucocyte counts in the blood were within the reference range. There were no clinical signs of inflammation in any of the udder quarters, and secretion was normal macroscopically and in the California mastitis test. The number of somatic cells in the milk was less than 100,000 cells/mL. On bacterial investigation, no pathogenic bacteria were found.

Group 2 consisted of 30 animals with clinical mastitis. All cows showed clinical signs of inflammation in one or more udder quarters and the secretion showed macroscopic alterations. The number of somatic cells in the milk of the infected udder quarter exceeded 100,000 cells/mL. In all of these cows, pathogenic bacteria could be found in the milk. All animals from group 2 needed to be treated. They received penethamate hydroiodide parenterally for three days and amoxicillin/clavulanic acid intra-mammarily for three to five milking periods.

Group 3 included 30 cows with subclinical mastitis. The diagnosis was based on the number of somatic cells, which exceeded 100,000 cells/mL in milk. There were no clinical signs of inflammation in any of the udder quarters, and secretion showed no alteration macroscopically or in the California mastitis test. These animals were not treated with antibiotics.

All cows from groups 2 and 3 were sampled two times. The first sampling was performed on day one, with the onset of signs in group 2 and the recognition of increased cell count of milk in group 3. The second sampling was performed on day 12, which was after the treatment in the groups of clinical mastitis.

All investigations were authorised by the Lower Saxony State Office for Consumer Protection and Food Safety (Niedersächsisches Landesamt für Verbraucherschutz und Lebensmittelsicherheit, LAVES) (file number: 33.9-42502-05-16A089), performed in compliance with the Animal Welfare Act and supervised by the Animal Welfare Officer of the Georg-August-University Göttingen (Germany).

### 2.2. Samples

#### 2.2.1. Milk: Preparation for PCT Measurement, Somatic Cell Count, and Bacteriology

Prior to collecting the milk samples, each cow’s teats were thoroughly cleaned with moist cleansing wipes that were pH neutral to the skin. In group 1, milk samples were taken from every quarter of each cow. For group 2, all inflamed quarters of the udder were sampled, but for group 3, only the quarter showing the highest somatic cell count according to the California mastitis test was used for sampling.

We used a part of the milk sample for PCT analysis. First, the milk was centrifuged for 15 min at 1000× *g*. After removing the fat layer, sample supernatant was pipetted off, aliquoted into Eppendorf tubes, and stored frozen at −80 °C.

A second part of the milk sample was taken for somatic cell measurement using automated optical fluorescence counting. In total, 5 mL of the sampled milk was placed into specially developed tubes for mastitis diagnostics (IfM, Verden, Germany) before being sent to the Institute for Milk Testing (Institut für Milchuntersuchung, IfM, Verden, Germany). If the milk’s character was no longer present as occurred in a few cases in this study and the milk consisted almost entirely of lumpy flocs, the somatic cell count could not be measured.

Using a third part of the milk sample, microbiological tests were carried out at the Institute of Microbiology at the University of Veterinary Medicine Hannover (Germany) by applying 10 µL of the sample to different culture media which included Columbia agar, Gassner agar, boiled blood agar, and selective agar for Staphylococcus and Streptococcus species and transferring 200 µL to a nutrient broth. Incubation was performed under aerobic conditions at 37 °C for 48 h, except for boiled blood agar, which was incubated under microaerophilic conditions. Bacteria were differentiated either by mass spectrometry or by culture biochemistry and serology. The colonization levels were defined as low if 1.0 × 10^3^ cfu/mL milk, medium as 1.0 × 10^4^ to 10^5^ cfu/mL milk, and high if exceeding 1.0 × 10^6^ cfu/mL milk.

#### 2.2.2. Blood: Preparation for PCT Measurement, Hematology, and Clinical Chemistry

Blood samples were collected out of the median caudal vein with a sterile 20 G 0.9 × 40 mm injection cannula and placed into serum and EDTA tubes (Sarstedt AG & Co, Nümbrecht, Germany). Of the serum tubes, one was centrifuged for 15 min at 1000× *g* and then pipetted, aliquoted, and stored frozen at −80 °C until PCT analysis. The blood tests were performed for the detection of leucocytes as well as their differentiation and the measurement of biochemical parameters to determine the animals’ state of health. To exclude the possibility that PCT was influenced by the calcium concentration, serum calcium levels were also measured. The haematological analyses of the EDTA blood were carried out on the analysis machine IDEXX ProCyte DxTM (IDEXX Laboratories Inc., Westbrook, ME, USA). For the biochemical parameters, serum was centrifuged for 6 min at 1000× *g* and measured photometrically by Konelab 20i (Thermo Fischer Scientific Inc., Dreieich, Germany).

### 2.3. Detection of PCT by Enzyme-Linked Immunosorbent Assay (ELISA)

To measure the PCT content in serum and milk, a species-specific quantitative competitive ELISA was used (MyBioSource, San Diego, CA, USA, Cat. No.: MBS742222-96) with a reported inter- and intra-assay coefficient of variability of less than 10%. The intra-assay coefficient of variability was validated using readings from five milk samples and was confirmed as a CV% of 7%.

Each sample and standard were placed in triplicate on a PCT-specific polyclonal antibody pre-coated plate. A specific PCT antigen which is conjugated to horseradish pe-oxidase was added, and the plate was then incubated for one hour at 37 °C. After the plate was washed five times using wash solution, all the liquid was drained off, and then hydrogen peroxide and tetramethylbenzidine were added before the plate was incubated a second time at 37 °C for 15 min. To stop the reaction, sulphuric acid was added. The optical density was determined by photometry using a TECAN microplate reader (TECAN Austria GmBH, Grödig, Austria) at a wavelength of 450 nm. Based on the measured absorption of the standards, a four-parameter logistic curve was generated as a standard curve to calculate the PCT concentrations.

### 2.4. Statistics

To determine the group size, a power analysis was performed with G*Power 3.1.9.7 (University of Kiel, 2020). The coefficient of determination R2 was assumed to be 0.2, the alpha level 0.5, and the power 0.9. The resulting group sizes of 25–30 animals per group were used in the study.

The data were statistically analysed and plotted using Prism 8 (GraphPad Software, San Diego, CA, USA). The values were tested for normal distribution using the Shapiro–Wilk test. Median, lower and upper quartile, maximum, minimum, and mean values were displayed through box and scatter plots, and the standard deviation was calculated. Comparisons were made between abnormal distributed values.

Serum and milk concentrations of PCT and cells/mL were compared between the groups using the Mann–Whitney U test as a test used for comparison of two independent groups.

The Wilcoxon signed-rank test as a test for two interdependent groups was used for the initial and re-examination of the same animal.

*p*-value less than 0.05 was considered statistically significant.

Furthermore, receiver operation characteristics (ROC) analysis was conducted for the determination of the cut-off value for the differentiation between subclinical mastitis and healthy cows.

## 3. Results

### 3.1. Comparison of PCT Concentrations in Healthy Cows vs. Cows with Clinical Mastitis

Twenty-eight clinically healthy cows in group 1 were analysed and sampled with a median somatic cell count in milk of 41,000 cells/mL, ranging from 7000 to 100,000 cells/mL. The animals in this group showed no signs of any disease, had no pyrexia, and the blood leukocyte counts were within the normal range. No pathogenic bacteria were detected in the milk samples (Table 1). The serum concentrations of PCT detected in this group ranged from 0 to 2217 pg/mL with a median of 1166 pg/mL. In the milk, the PCT concentrations ranged from 0 to 832 pg/mL with a median of 167 pg/mL.

**Figure 1:**
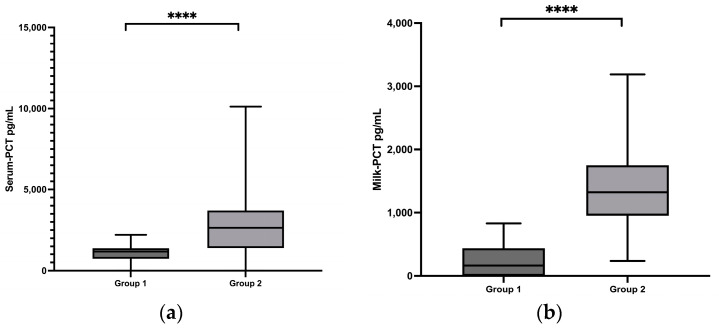

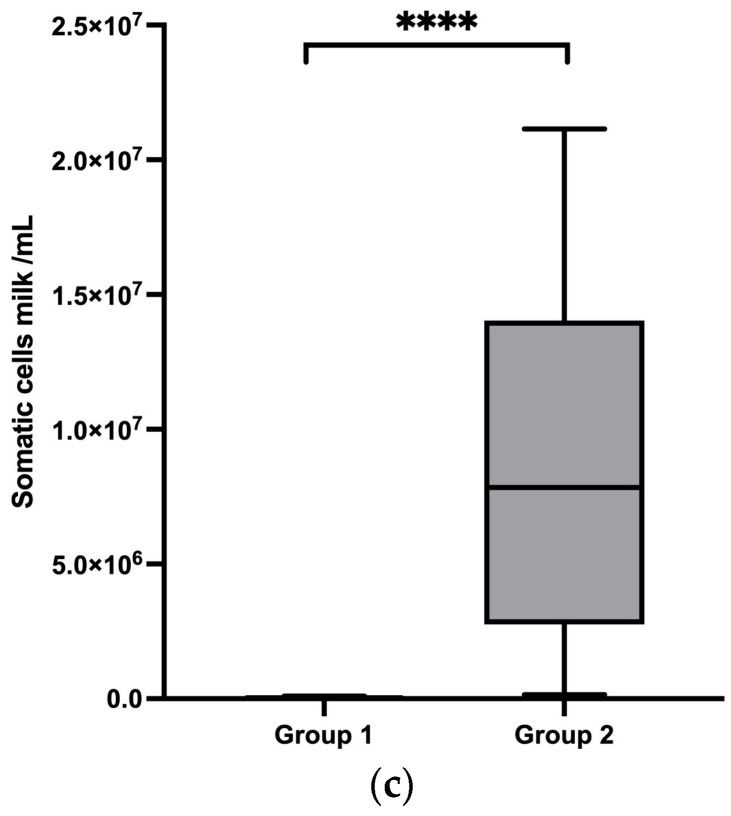
(**a**) Serum procalcitonin (PCT) concentrations in group 1 and group 2; (**b**) milk PCT concentrations in group 1 and group 2; (**c**) cell count in the milk in group 1 and group 2. Significant changes are marked by asterisks (*): **** *p* < 0.0001. The significance level is set at *p* < 0.05. Upper whisker = maximum; lower whisker = minimum; upper box line = 3rd quartile; middle box line = median; lower box line = 1st quartile.

Samples were then obtained from 30 cows in group 2 with clinical mastitis. Due to strong changes in the milk composition, the exact somatic cell count of the isolated affected udder quarter and statistical evaluations could only be measured in 14 cows. The median somatic cell count was 7,842,000 cells/mL, ranging from 155,000 to 21,142,000 cells/mL, which was significantly higher than in group 1 (*p* < 0.0001) (Figure 1c). In group 2, all cows exhibited characteristic signs of mastitis, and typical mastitis pathogens were detected by bacteriological examination in most of the samples, including Streptococcus uberis (*S*. *uberis*) in twelve samples and Escherichia coli (*E*. *coli*) in six samples, which were identified as the main pathogens (Table 1). The serum concentrations of PCT in group 2 ranged from 0 to 10,121 pg/mL, with a median of 2641 pg/mL (Figure 1a). In the milk concentrations of PCT, between 239 and 3186 pg/mL with a median of 1326 pg/mL was measured. The PCT serum and milk concentrations in group 2 were significantly higher than in the healthy control group (*p* < 0.0001) (Figure 1b).

The study found no correlations between PCT concentrations and mastitis pathogens or internal body temperature and blood leukocyte number.

After 12 days, we re-examined all cows in group 2. There was a significant decrease in the number of cells from initial examination to follow up (*p* = 0.0105), taking into account only the measurable somatic cell counts. A median cell count of 701,000 cells/mL was found in the milk of 28 measurable samples, ranging from 56,000 to 21,628,000 cells/mL. Seven cases of *S*. *uberis* and *E*. *coli* were still detected. Serum PCT concentrations ranged from 671 to 14,251 pg/mL, with a median of 2718 pg/mL. In milk, concentrations ranged from 0 to 3415 pg/mL with a median of 1652 pg/mL. No significant difference in PCT serum and milk concentrations was detectable between the initial and repeated testing (*p* > 0.5).

### 3.2. Comparison of PCT Concentrations between Healthy Cows and Cows with Subclinical Mastitis

**Figure 2:**
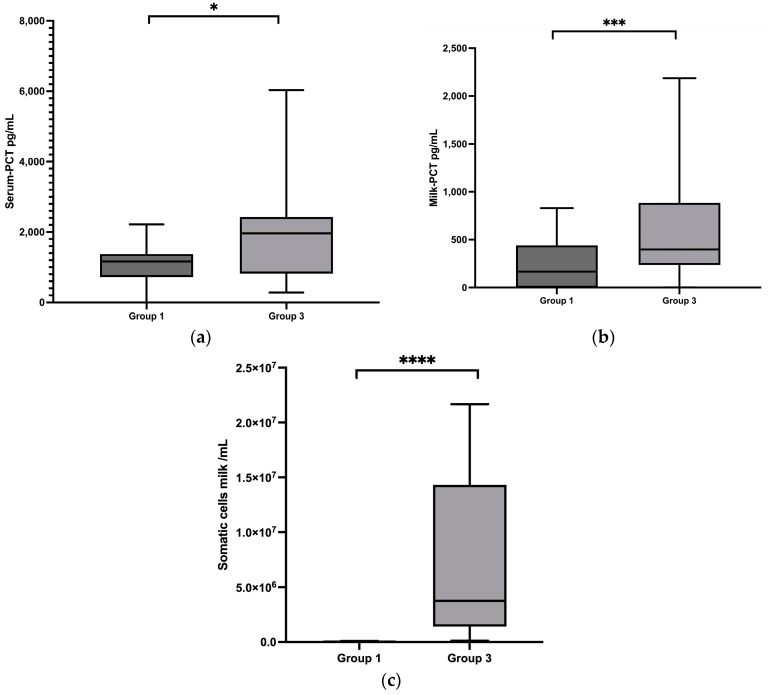
(**a**) Serum procalcitonin (PCT) concentrations in group 1 and group 3; (**b**) milk PCT concentrations in group 1 and group 3; (**c**) cell count in the milk in group 1 and group 3. Significant changes are marked by asterisks (*): * *p* < 0.05, *** *p* < 0.001, **** *p* < 0.0001. The significance level is set at *p* < 0.05. Upper whisker = maximum; lower whisker = minimum; upper box line = 3rd quartile; middle box line = median; lower box line = 1st quartile.

**Figure 3:**
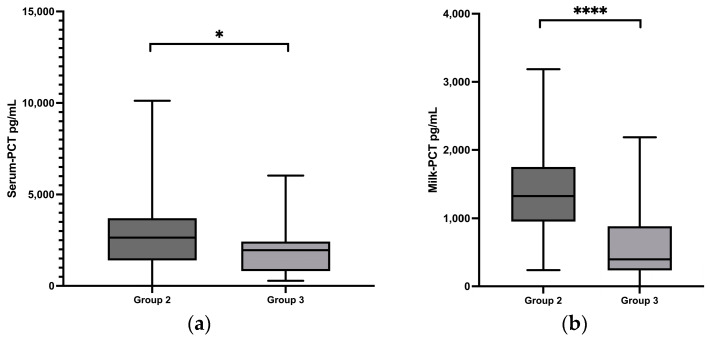

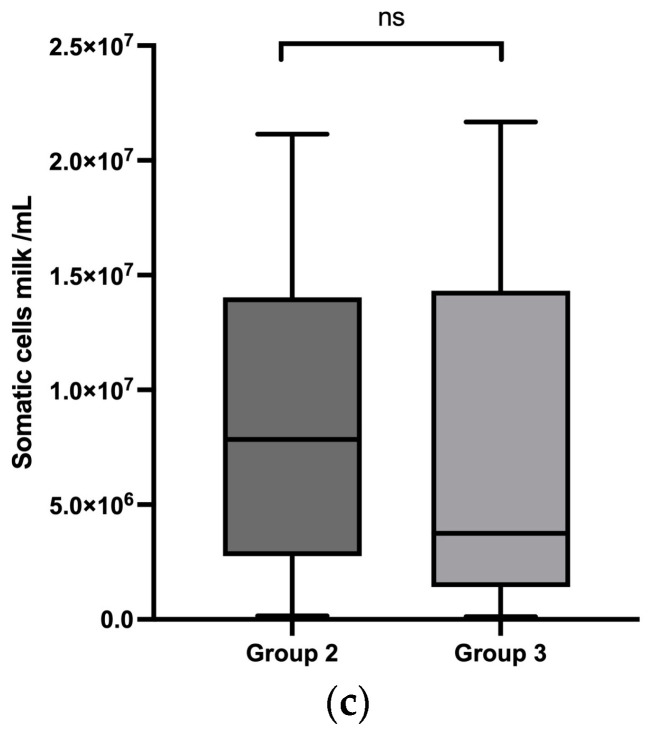
This is a figure. (**a**) Serum procalcitonin (PCT) concentrations in group 2 and group 3; (**b**) milk PCT concentrations in group 2 and group 3; (**c**) cell count in the milk in group 2 and group 3. Significant changes are marked by asterisks (*): * *p* < 0.05, **** *p* < 0.0001. ns means “not significant”, *p* > 0.05. The significance level is set at *p* < 0.05. Upper whisker = maximum; lower whisker = minimum; upper box line = 3rd quartile; middle box line = median; lower box line = 1st quartile.

The PCT concentrations in group 1 healthy cows were compared those in group 3 cows diagnosed with subclinical mastitis. These cows had no clinical signs of mastitis but showed an increased somatic cell count with a median value of 3,753,000 cells/mL ranging from 117,000 to 21,673,000 cells/mL (Table 1), which was significantly higher than in group 1 (*p* < 0.0001) (Figure 2c), but being not significantly lower compared to the mean value in group 2 (*p* > 0.05) (Figure 3c). Pathogenic bacteria were found in milk including *S*. *uberis* in eleven samples, *S. aureus* in seven samples, and *S*. *dysgalactiae* in five samples. Serum PCT concentrations ranged from 282 to 6033 pg/mL, with a median of 1961 pg/mL, and in milk, concentrations ranged between 0 and 2186 pg/mL, with a median of 396 pg/mL. Both the serum (*p* = 0.0343) (Figure 2a) and milk (*p* = 0.0007) (Figure 2b) concentrations of PCT in group 3 were significantly higher than those in group 1.

### 3.3. Comparison of PCT Concentrations between Cows with Clinical Mastitis and Cows with Subclinical Mastitis

Both the serum (*p* = 0.0246) (Figure 3a) and milk (*p* < 0.0001) (Figure 3b) PCT concentrations in group 3 were significantly lower than those in group 2. There were no significant correlations between PCT concentrations and mastitis pathogens or internal body temperature and leukocyte number in the blood.

### 3.4. Comparison of Cells and PCT in Groups 2 and 3 between the Initial Examination and a Re-Examination after 12 Days

**Figure 4:**
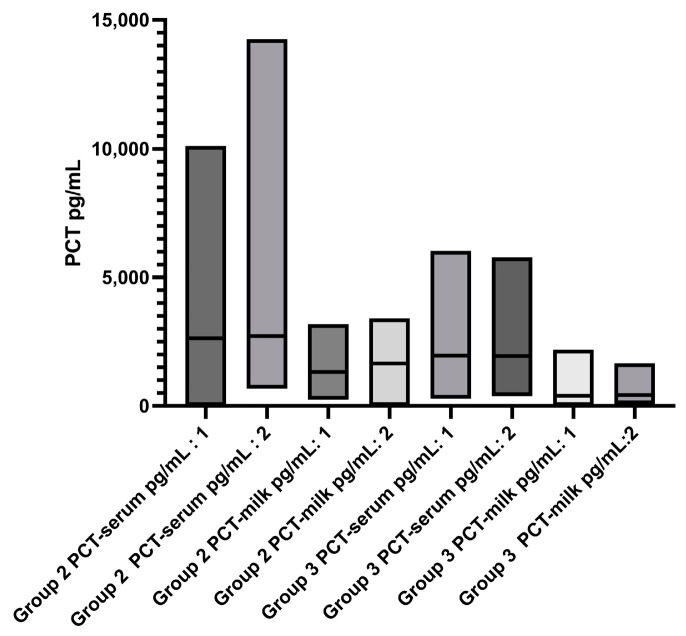
Serum and milk PCT concentrations and the initial examination (:1) and after 12 days (:2).

After 12 days, all animals of groups 2 and 3 underwent re-examination, and no animal exhibited signs of clinical mastitis. There was no significant difference between the somatic cell count at baseline and re-examination. Five samples with *S*. *uberis*, seven samples with *S*. *aureus*, three samples with *S*. *dysgalactiae*, and two samples with *E*. *coli* were still detected. There was no significant difference in the PCT levels in the serum and milk of both groups (group 2 and group 3) between the initial examination and the re-examination after 12 days (Figure 4).

### 3.5. ROC Analysis

**Figure 5:**
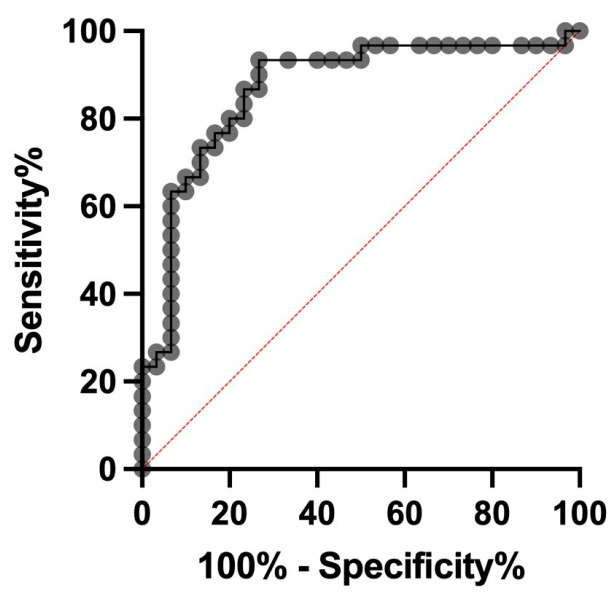
ROC analyses. To differentiate cows with clinical mastitis from those with subclinical mastitis, the PCT measurement in milk can be used with a sensitivity of 0.80 and a specificity of 0.8 at a cut-off of 899 pg/mL. The AUC is 0.87.

In order to differentiate cows with subclinical mastitis from healthy controls, an ROC curve was generated. A sensitivity of 0.66 and a specificity of 0.35 were calculated for serum PCT at a cut-off of 886 pg/mL, with an area under the curve (AUC) of 0.66. For milk PCT, a sensitivity of 0.76 and a specificity of 0.64 were computed at a cut-off of 234.5 pg/mL, with an AUC of 0.75. To differentiate cows with clinical mastitis from those with subclinical mastitis, the PCT measurement in the milk can be used with a sensitivity of 0.80 and a specificity of 0.80 at a cut-off of 899 pg/mL. The AUC is 0.87 (Figure 5).

## 4. Discussion

In human medicine, there has already been a move to seek objective biomarkers to complement subjective clinical examination and to help decide on required therapy. The use of antibiotics is rising [13], with the result that the development of antibiotic resistance in bacteria is increasing [14]. Procalcitonin could be a useful marker, because its concentrations increase during bacterial infections and remain elevated through to recovery [3]. Apart from serum, PCT has already been measured in human saliva [15], in human wound exudate [16], and in human breastmilk [17].

Only a few studies exist on the use of PCT in veterinary medicine [3]. Based on its clinical use in human medicine, the use of PCT as a biomarker of progression and prognosis in veterinary medicine would be desirable. In our study, we investigated PCT in cows with mastitis. Mastitis is an important disease because it is very common in cows, results in severe financial losses [18], and is usually treated with antibiotics [19]. In this study, PCT concentrations of the serum and milk in healthy control cows were compared to those of cows with subclinical and clinical mastitis. In addition, levels of PCT concentration from a baseline examination and a re-examination twelve days later were compared. Our results showed that PCT is measurable in the serum and milk of cows. In another study on mastitis in cows, PCT was only measurable in plasma but not in milk using a commercial ELISA [20]. This indicates that the use of a commercial ELISA for the measurement of PCT in samples other than serum or plasma may be difficult.

The results also showed that PCT levels in cows with clinical mastitis were significantly higher than in healthy cows, confirming the theory that PCT increases in bacterial infections [21]. Because the clinically ill animals were sampled immediately after the onset of signs and PCT levels were already greatly elevated at this time, PCT was confirmed as an early marker of infection [22]. These results were confirmed in a study on cow mastitis caused by Staphylococcus. Elevated PCT concentrations were also measured in serum in this study [9].

The PCT concentrations in serum and milk in cows with subclinical mastitis were also significantly higher than those of the healthy control group. Compared to the clinical mastitis group, the serum and milk PCT levels were significantly lower in both milk and serum, falling into an intermediate range between clinical mastitis and healthy cows. The differences in PCT concentrations between each of the three groups were more significant in milk than in serum. Another study that compared PCT systemically in serum with a local medium was published by Barton et al. [23], where PCT in horses with chronic pneumonia was examined in serum and bronchoalveolar lavage fluid (BALF), concluding that the measurement in serum rather than in BALF was more significant.

This study showed that PCT levels correlated with the type of mastitis and that PCT measurements could be used to better select the type and duration of appropriate therapy [24,25]. In PCT-guided, randomized trials by Christ-Crain et al. [26], only 44% of subjects in a study group received antibiotics based on the PCT concentration in their blood, compared with 83% of human patients in the control group treated according to guidelines. There were no obvious differences in recovery, and based on PCT concentrations, the duration of therapy was reduced on average from 12 to 5 days. The same principles applied to this study could mean that medium PCT levels in cows with subclinical mastitis could be considered a group where antibiotics should not be used immediately for treatment, but observation of the affected animals should continue. In cases of clinical mastitis with highly elevated PCT levels, direct use of antibiotics would be reasonable.

The results of our ROC analyses showed that the discrimination between clinical mastitis requiring treatment with antibiotics and subclinical mastitis can be based on the PCT concentration in milk. At a cut-off of 899 pg/mL, the two diseases can be distinguished with a sensitivity and a specificity of 80%. Measurement in milk makes the use of PCT under practical conditions particularly easy.

According to results reported in the literature, which states that mastitis should be treated for three to eight days [27,28], the timing of re-examination in this study was determined. The cows with clinical and subclinical mastitis in this study still have significantly elevated PCT concentrations in milk and blood compared to the healthy control group even 12 days after the onset of signs. This contradicts the statement of Assicot et al. [21] that PCT decreases rapidly after antibiotic therapy. One hypothesis was that the animals were still not fully recovered from the infection, which is supported by the somatic cell count in the milk, which had decreased on average but remained clearly above the reference of 100,000 cells/mL, and 10% of the clinically ill animals still showed poor general condition. Regardless of antibiotic therapy, PCT concentrations in the serum of humans in the study by Preas et al. [29] peaked after 24 h following a single injection of endotoxic lipopolysaccharides and remained elevated for at least 7 days, sometimes not returning to normal for up to 2 weeks. Even 7 weeks after clinical infection, the animals in this kinetics study still showed elevated PCT concentrations in milk and serum, which, although steadily decreasing, were still higher than the median levels in the healthy control group. These results were consistent with those of Fogsgaard et al. [30], who reported that cows with clinical mastitis remained in a state of incomplete recovery even after 8 weeks of antibiotic therapy. When the somatic cell count results are considered as the standard for detecting and monitoring mastitis, it is noticeable that they decrease much faster than PCT concentrations, so PCT would seem to be more appropriate for predicting complete recovery after clinical mastitis, because the course of the disease seems to last longer in clinical and subclinical mastitis than previously thought.

There were no significant correlations between PCT concentrations and variables including somatic cell count, internal body temperature, and leukocyte number. The lack of correlation between PCT and leukocytes may be related to the fact that relatively low expression of PCT has been detected in white blood cells [31], indicating a more tissue-based rather than leukocyte-based defence system [32]. The current standard for the detection and assessment of the disease course of mastitis is the measurement of somatic cell count, but this cannot always be accurately determined due to various parameters, such as the alteration of pH values or the precipitation of proteins, and may therefore not always be accurate. Furthermore, somatic cell count is not able to differentiate between clinical and subclinical mastitis, whereas PCT was able to do so in this study.

The pathogen type identified as the cause of clinical mastitis was compared to PCT concentrations. The main pathogens were divided into streptococci, staphylococci, and E. coli. There was a tendency for the highest PCT concentrations in milk to be found in *E*. *coli* mastitis, which is often particularly severe, but serum PCT concentrations tended to be the lowest. None of the comparisons were significant, and the reason why PCT values in milk and serum are so different remains unclear. Because the sample size of six cows with *E*. *coli* mastitis was very small, further studies with a larger sample size are needed, and it is possible that significant differences between the bacterial groups might emerge. However, because no significant correlation was found in this study, it must be assumed that PCT increases independently of the pathogen in the case of a bacterial infection. The distribution of pathogens found in this study corresponded to that found in other studies [33,34].

No animals were diagnosed with hypercalcemia in our study, so the possibility that the increase in PCT was based on hypercalcemia and increased calcitonin was excluded, supporting Barton et al. [23], who indicated that PCT increased independently of serum calcium levels during bacterial infections. This proved that PCT was not only important as a precursor protein of calcitonin responsible for regulating blood calcium levels but also had its own role in the body independent of calcium, when bacterial infections occur. Elevated PCT levels are also potentially harmful [32,35], which could lead to a new approach treating not only with antibiotics but also with PCT immune neutralization.

The main limitation of this study is the relatively low number of cows per group. Future studies based on larger groups are needed to confirm our results.

## 5. Conclusions

The results of the present study show that the PCT concentration in blood and milk of cows with acute and subclinical mastitis is elevated; thus, the two diseases can be distinguished from healthy cows by the PCT concentration. However, this is also possible when the cell count in the milk is measured. The latter, however, is less suitable for distinguishing subclinical from clinical mastitis. However, PCT concentration in both blood and milk is suitable to distinguish subclinical mastitis from acute mastitis. Against this background, the measurement of PCT seems to be better suited to distinguish mastitis of different severity and course than is possible with currently established methods. This may have an impact on the therapeutic options in mastitis including the administration of antibiotics.

However, PCT is not specific for inflammation of the udder. In particular, for PCT measurement in blood, inflammatory diseases of other organs must be excluded clinically or by laboratory diagnosis.

## Figures and Tables

**Table 1:** Summary of clinical and laboratory signs of each group.

	Cows with Clinical Mastitis		Cows with Subclinical Mastitis		Healthy Control Cows T1
	T1	T2	T1	T2	
Internal body Temperature (°C)	39.3 (38.3 to 40.8)	38.7 (38.1 to 41.1)	38.4 (37.6 to 38.9)	38.6 (37.8 to 38.9)	38.3 (37.3 to 38.9)
Somatic cells/mL (*n* = measurable samples)	7,842,000 (*n* = 14) (155,000 to 21,142,000)	701,000 (*n* = 28) (56,000 to 21,628,000)	3,753,000 (*n* = 30) (117,000 to 21,673,000)	1,206,000 (*n* = 29) (101,000 to 23,305,000)	47,000 (*n* = 28) (7000 to 100,000)
Leukocytes (10^3^/µL)	6.9 (*n* = 24) (0.4 to 13)	9.0 (*n* = 30) (0.2 to 27.5)	8.7 (*n* = 29) (5.4 to 17.9)	7.6 (*n* = 29) (3.5 to 11.4)	7.3 (*n* = 27) (4.7 to 10.0)
Bacteria (main pathogens)	*Streptococcus uberis* (*n* = 12), *Escherichia coli* (*n* = 6)	*Streptococcus uberis* (*n* = 7), *Escherichia coli* (*n* = 7)	*Streptococcus uberis* (*n* = 11), *Staphylococcus aureus* (*n* = 7), *Streptococcus dysgalactiae* (*n* = 5)	*Streptococcus uberis* (*n* = 5) *Staphylococcus aureus* (*n* = 7), *Streptococcus dysgalactiae* (*n* = 3), *Escherichia coli* (*n* = 2)	*-*

T1 marks the initial time of sampling; T2 the time of sampling after 12 days.

## Data Availability

Data sharing is not applicable.
